# Complete genome sequence of the potato pathogen *Ralstonia solanacearum* UY031

**DOI:** 10.1186/s40793-016-0131-4

**Published:** 2016-01-15

**Authors:** Rodrigo Guarischi-Sousa, Marina Puigvert, Núria S. Coll, María Inés Siri, María Julia Pianzzola, Marc Valls, João C. Setubal

**Affiliations:** Instituto de Química, Universidade de São Paulo, São Paulo, Brazil; Department of Genetics, University of Barcelona and Centre for Research in Agricultural Genomics (CRAG), Bellaterra, Catalonia, Spain; Departamento de Biociencias, Cátedra de Microbiología, Facultad de Química, Universidad de la República, Montevideo, Uruguay; Biocomplexity Institute, Virginia Tech, Blacksburg, VA USA

**Keywords:** Short genome report, Bacterial wilt, *Ralstonia solanacearum*, Bacterial plant pathogen, Methylome, Uruguay

## Abstract

*Ralstonia solanacearum* is the causative agent of bacterial wilt of potato. *Ralstonia solanacearum* strain UY031 belongs to the American phylotype IIB, sequevar 1, also classified as race 3 biovar 2. Here we report the completely sequenced genome of this strain, the first complete genome for phylotype IIB, sequevar 1, and the fourth for the *R. solanacearum* species complex. In addition to standard genome annotation, we have carried out a curated annotation of type III effector genes, an important pathogenicity-related class of genes for this organism. We identified 60 effector genes, and observed that this effector repertoire is distinct when compared to those from other phylotype IIB strains. Eleven of the effectors appear to be nonfunctional due to disruptive mutations. We also report a methylome analysis of this genome, the first for a *R. solanacearum* strain. This analysis helped us note the presence of a toxin gene within a region of probable phage origin, raising the hypothesis that this gene may play a role in this strain’s virulence.

## Introduction

*Ralstonia solanacearum* is the causal agent of bacterial wilt, one of the most devastating plant diseases worldwide [1]. It is a highly diversified bacterial plant pathogen in terms of host range, geographical distribution, pathogenicity, epidemiological relationships, and physiological properties [2]. Strains are divided in four phylotypes, corresponding roughly to their geographic origin: Asia (phylotype I), the Americas (II), Africa (III), and Indonesia (IV) [3]. Strain UY031 belongs to phylotype IIB, sequevar 1 (IIB1), the group considered mainly responsible for bacterial wilt of potato in cold and temperate regions [4]. Phylotype IIB, sequevar 1 is also traditionally classified as race 3 biovar 2.

Strain UY031 was isolated in Uruguay from infected potato tubers in 2003 and displays high aggressiveness both on potato and tomato hosts [5]. This strain is being used as a model in plant-pathogen gene expression studies carried out by our group; having its genome available greatly facilitates the identification of pathogenicity-related genes. Four other IIB1 *R. solanacearum* strains have been partially sequenced: UW551 [6], IPO1609 [7], NCPPB909 [8], and CFIA906 [8]. This is the first genome of this group to be completely sequenced, and the fourth within the *R. solanacearum* species complex (the other three are strains GMI1000 [9], Po82 [10] , and PSI07 [11]).

## Organism information

### Classification and features

**Fig. 1:**
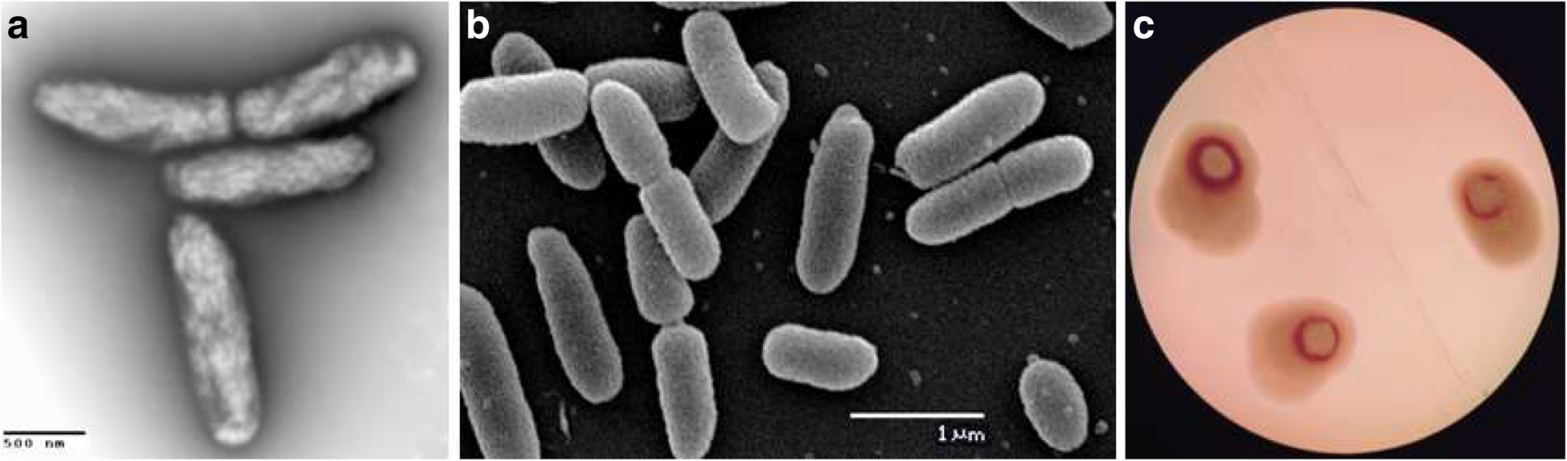
Images of *Ralstonia solanacearum* strain UY031 using transmission (**a**) and scanning (**b**) electron microscopy, as well as light microscopy to visualize colony morphology on solid media (**c**)

**Fig. 2:**
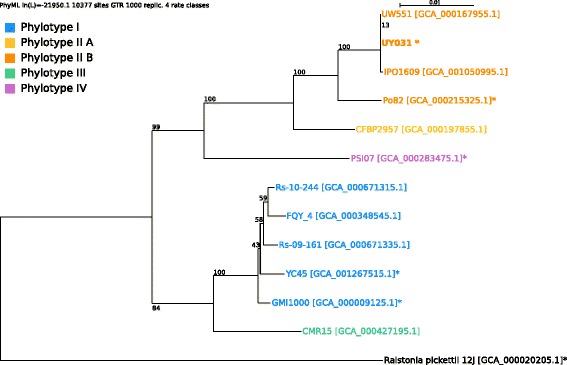
Phylogenetic tree highlighting the position of the *Ralstonia solanacearum* UY031 (shown in bold) relative to other strains from the same species. The phylogenetic tree was constructed using four conserved prokaryotic marker genes, namely: recA, rpoA, rpoB and rpoC. Each gene was aligned individually with MUSCLE [25]; the resulting multiple alignments were concatenated. PhyML [26] was used to perform tree reconstruction using the GTR model and 1,000 bootstrap replicas. Strain names are colour-coded according to the correspondent phylotype. GenBank accession numbers are displayed within brackets. Strains whose genome was completely sequenced are marked with an asterisk. *Ralstonia pickettii* 12 J (NCBI accession NC_010682) was used as an outgroup

*Ralstonia solanacearum* UY031 strain is classified within the order *Burkholderiales* of the class *Betaproteobacteria*. It is an aerobic, non-sporulating, Gram-negative bacterium with rod-shaped cells ranging from 0.5 to 1.5 μm in length (Fig. 1, (a) and (b)). The strain is moderately fast-growing, forming 3**–**4 mm colonies within 2**–**3 days at 28 **°**C. On a general nutrient medium containing tetrazolium chloride and high glucose content, strain UY031 usually produces a diffusible brown pigment and develops pearly cream-white, flat, irregular, and fluidal colonies with characteristic pink whorls in the centre (Fig. 1, (c)). Strain UY031 was isolated from a naturally infected potato tuber showing typical brown rot symptoms (creamy exudates from the vascular rings and eyes of the tuber). This strain is highly pathogenic in different solanaceous hosts including important crops like tomato and potato [5]. Pathogenicity of this strain was also confirmed in several accessions of *Solanum commersonii* Dunal, a wild species considered as a valuable source of resistance for potato breeding. Due to its great aggressiveness, strain UY031 is being used for selection of resistant germplasm as part of the potato breeding program developed in Uruguay. This strain has been deposited in the CFBP collection of plant-associated bacteria, and has received code CFBP 8401. Minimum Information about the Genome Sequence of *R. solanacearum* strain UY031 is summarized in Table 1, and a phylogenetic tree is shown in Fig. 2.

**Table 1:** Classification and general features of *Ralstonia solanacearum* strain UY031according to the MIGS recommendations [27]

MIGS ID	Property	Term	Evidence code^a^
	Classification	Domain Bacteria	TAS [28]
		Phylum *Proteobacteria*	TAS [29]
		Class *Betaproteobacteria*	TAS [30, 31]
		Order *Burkholderiales*	TAS [31, 32]
		Family *Burkholderiaceae*	TAS [31, 33]
		Genus *Ralstonia*	TAS [34, 35]
		Species *Ralstonia solanacearum*	TAS [34, 35]
		Strain UY031	
	Gram stain	Negative	IDA
	Cell shape	Rod	IDA
	Motility	Motile	IDA
	Sporulation	Non sporulating	NAS
	Temperature range	Mesophile	IDA
	Optimum temperature	27 °C	IDA
	pH range; Optimum	5.5 – 8.0; 6.5	NAS
	Carbon source	Dextrose, lactose, maltose, cellobiose	IDA
MIGS-6	Habitat	potato plants, soil	TAS [5]
MIGS-6.3	Salinity	<2.0 %	TAS [36]
MIGS-22	Oxygen requirement	Aerobic	IDA
MIGS-15	Biotic relationship	free-living	IDA
MIGS-14	Pathogenicity	Pathogenic	TAS [5]
MIGS-4	Geographic location	Uruguay, San José	TAS [5]
MIGS-5	Sample collection	2003	TAS [5]
MIGS-4.1	Latitude	34°43′58.17”S	NAS
MIGS-4.2	Longitude	56°32′2.87”W	NAS
MIGS-4.4	Altitude	116.7 m	NAS

^a^Evidence codes - *IDA* Inferred from direct assay, *TAS* Traceable author statement (i.e., a direct report exists in the literature), *NAS* Non-traceable author statement (i.e., not directly observed for the living, isolated sample, but based on a generally accepted property for the species, or anecdotal evidence). These evidence codes are from the Gene Ontology project [37]

## Genome sequencing information

### Genome project history

This sequencing project was carried out in 2015; the result is a complete and finished genome. Project data is available from GenBank (Table 2). Accession codes for reads in the Sequence Read Archive are SRP064191, SRR2518086, and SRZ132405.

**Table 2:** Project information

MIGS ID	Property	Term
MIGS 31	Finishing quality	Finished
MIGS-28	Libraries used	SMRT library (P5-C3 large insert library)
MIGS 29	Sequencing platforms	PacBio RS II
MIGS 31.2	Fold coverage	138×
MIGS 30	Assemblers	HGAP.2 workflow
MIGS 32	Gene calling method	Prokka v1.10 (ncRNAs search enabled)
	Locus tag	RSUY
	Genbank ID	CP012687 (chr), CP012688 (pl)
	GenBank date of release	September 28, 2015
	GOLD ID	NA
	BIOPROJECT	PRJNA278086
MIGS 13	Source material identifier	SAMN03402637
	Project relevance	Plant pathogen

**Table 3:** Summary of genome: one chromosome and one plasmid

Label	Size (Mb)	Topology	INSDC identifier	RefSeq ID
Chromosome	3.41	circular	NA	NA
Megaplasmid	1.99	circular	NA	NA

### Growth conditions and genomic DNA preparation

*R. solanacearum* strain UY031 was routinely grown in rich B medium (10 g/l bactopeptone, 1 g/l yeast extract and 1 g/l casaminoacids). Genomic DNA was extracted from a bacterial culture grown to stationary phase to avoid over-representation of genomic sequences close to the origin of replication. Twelve ml of a culture grown for 16 h at 30 °C and shaking at 200 rpm (OD_600_ = 0.87) were used to extract DNA with Blood & Cell Culture DNA Midi kit (Qiagen), following manufacturer’s instructions for gram-negative bacteria. DNA concentration and quality were measured in a Nanodrop (ND-8000 8-sample spectrophotometer).

### Genome sequencing and assembly

Whole-genome sequencing was performed on the PacBio RS II platform at the Duke Center for Genomic and Computational Biology (USA). P5-C3 chemistry and a single SMRTcell were used, and quality control was performed with DUGSIM. The number of Pre-Filter Polymerase Read Bases was greater than 749 million (>130x genome coverage). Reads were assembled using RS_HGAP_Assembly.2 protocol from SMRT Analysis 2.3 [12]. This resulted in one circular chromosome (3,412,138 bp) and one circular megaplasmid (1,999,545 bp). These lengths are very similar to those of the corresponding replicons in *R. solanacearum* Po82, a IIB sequevar 4 strain, also a potato pathogen and which has also been completely sequenced [10]. The origin of replication was defined for both replicons based on the putative origin for reference strain GMI1000 [9].

An assembly quality assessment was performed before all downstream analyses. All reads were mapped back to the assembled sequences using RS_Resequencing.1 protocol from SMRT Analysis 2.3. This analysis revealed that chromosome and megaplasmid sequences had 100 % of bases called (percentage of assembled sequence with coverage > = 1) and 99.9999 % and 99.9992 %, respectively, of consensus concordance.

### Genome annotation

Genome annotation was done using Prokka [13] with the option for ncRNA search. Type III effectors of strain UY031 were identified and annotated in three steps: First, 17 of the T3Es from the *R. solanacearum* species complex [14] were identified based on the Prokka annotations. Second, the 15 T3Es annotated as “Type III Effector Protein”, “Probable Type III Effector Protein” or “Putative Type III Effector Protein” by Prokka were manually annotated using the first BLAST [15] hits (usually 100 % identity) of their DNA sequences against genome sequences of phylotype IIB strains MOLK2 and Po82. Third, the UY031 genome was uploaded to the “*Ralstonia* T3E” web interface tool [14] to search for additional T3Es not annotated as such with Prokka. The additional 28 T3E genes identified were manually annotated as above. Homologous Gene Group clustering was performed with get_homologues [16] using the orthoMCL program [17] and requiring a minimum sequence identity in BLAST query/subject pairs of 30 %.

The sequencing plataform used to assemble the genome (PacBio RS II) also gives kinectics information about the sequenced genome. The presence of a methylated base in the DNA template delays the incorporation of the complementary nucleotide; such modifications in the kinectics may be used to characterize modified bases by methylation including: 6-mA, 5-mC and 4-mC [18]. The analysis of these modifications in a genome-wide and single-base-resolution scale allowed us to characterize the ‘methylome’ of this strain. These epigenetic marks are commonly used by bacteria, and its implications vary from a defense mechanism, protecting the cell from invading bacteriophages or other foreign DNA, to the bacterial virulence itself [19–21]. We performed methylome analysis and motif detection using RS_Modification_and_Motif_analysis.1 protocol from SMRT Analysis 2.3. Such epigenetic marks arise from DNA methyl-transferases, sometimes coupled with a restriction endonuclease (a Restriction-Modification System). We further characterized which genes give rise to the modified motifs using tools available at REBASE [22].

## Genome properties

The genome of *R. solanacearum* strain UY031 has one chromosome (3,412,138 bp) and one circular megaplasmid (1,999,545 bp) (Table 3). The average GC content of the chromosome is 66.5 % while that of the megaplasmid is 66.7 %. A total of 4,778 genes (4,683 CDSs and 95 RNAs) were predicted. Of the protein-coding genes, 3,566 (76.1 %) had functions assigned while 1,212 were considered hypothetical (Table 4). Of all CDSs, 76.6 % could be assigned to one COG functional category and for 83.1 % one or more conserved PFAM-A domains were identified (Table 5).

**Table 4:** Genome statistics

Attribute	Value	% of total
Genome size (bp)	5,411,683	100.00
DNA coding (bp)	4,737,274	87.5
DNA G + C (bp)	3,604,487	66.6
DNA scaffolds	2	100.00
Total genes	4,778	100.00
Protein coding genes	4,683	98.0
RNA genes	95	1.9
Pseudo genes	NA	NA
Genes in internal clusters	NA	NA
Genes with function prediction	3,566	74.6
Genes assigned to COGs	3,586	76.6
Genes with Pfam domains	3,892	83.1
Genes with signal peptides	501	10.6
Genes with transmembrane helices	1132	24.1
CRISPR repeats	0	-

**Table 5:** Number of genes associated with general COG functional categories

Code	Value	%	Description
J	160	3.4	Translation, ribosomal structure and biogenesis
A	2	<0.1	RNA processing and modification
K	273	5.8	Transcription
L	240	5.1	Replication, recombination and repair
B	3	<0.1	Chromatin structure and dynamics
D	28	0.6	Cell cycle control, Cell division, chromosome partitioning
V	45	1.0	Defense mechanisms
T	162	3.5	Signal transduction mechanisms
M	237	5.1	Cell wall/membrane biogenesis
N	119	2.5	Cell motility
U	61	1.3	Intracellular trafficking and secretion
O	154	3.3	Posttranslational modification, protein turnover, chaperones
C	226	4.8	Energy production and conversion
G	165	3.5	Carbohydrate transport and metabolism
E	342	7.3	Amino acid transport and metabolism
F	75	1.6	Nucleotide transport and metabolism
H	154	3.3	Coenzyme transport and metabolism
I	177	3.8	Lipid transport and metabolism
P	176	3.8	Inorganic ion transport and metabolism
Q	73	1.6	Secondary metabolites biosynthesis, transport and catabolism
R	352	7.5	General function prediction only
S	362	7.7	Function unknown
-	1097	23.4	Not in COGs

The total is based on the total number of protein coding genes in the genome

## Insights from the genome sequence

**Fig. 3:**
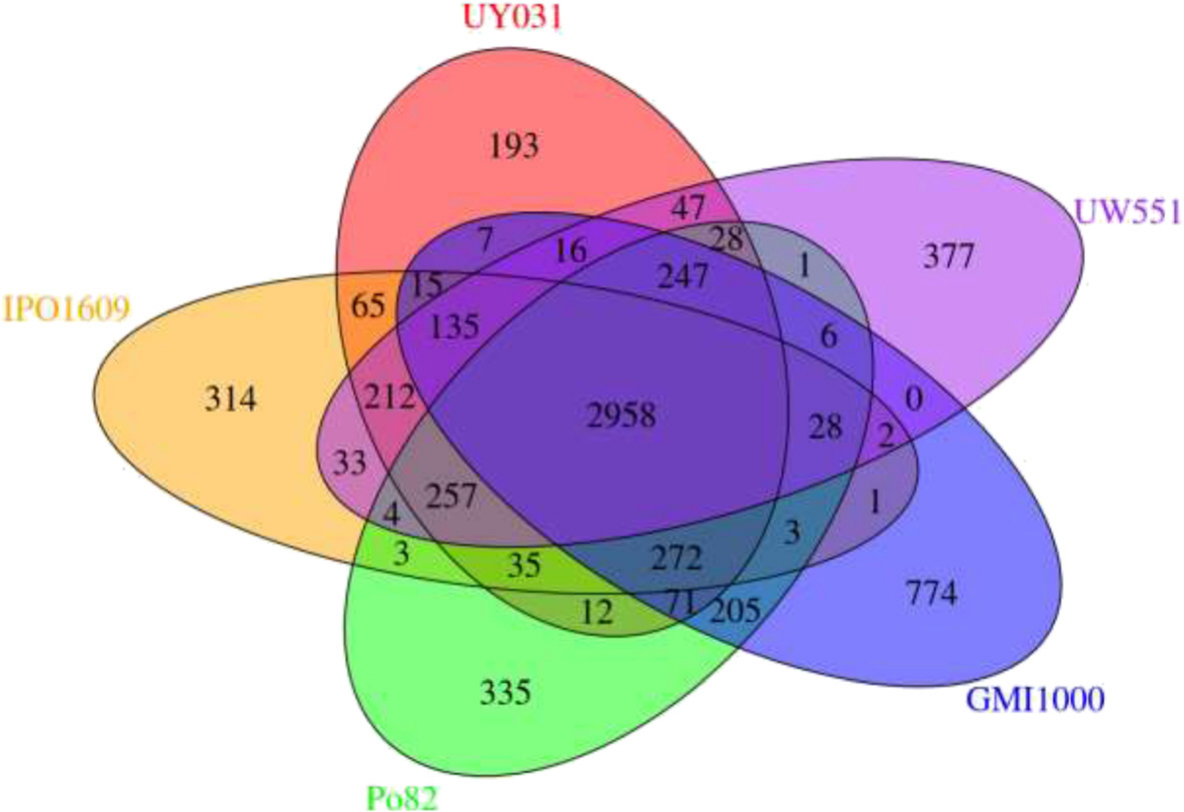
Venn diagram of the *Ralstonia solanacearum* homologous gene groups. The *R. solanacearum* genomes compared were as follows: strains Po82, GMI1000, IPO1609, UW551, and UY031

We performed a pan-genome analysis of the *R. solanacearum* UY031 genome, comparing it to four other genomes: two closely-related *R. solanacearum* strains (UW551 and IPO1609) and two others with complete genome sequences available (GMI1000 and Po82). The pan-genome consists of 7,594 HGGs while the core genome consists of 2,958 HGGs; the variable genome consists of 2,643 HGGs, and the number of strain-specific HGGs ranges from 193 to 774 (Fig. 3). We identified 193 HGGs that are UY031-specific; 75.1 % of them were annotated as hypothetical proteins.

Type III effector genes are among the most important for virulence determinants in bacterial plant pathogens such as *R. solanacearum* [14]*.* Based on comparisons with effector gene sequences in public databases (see above) we have identified 60 T3Es (Table 6), of which 11 appear to be nonfunctional due to frameshifts or other mutations that disrupt the coding sequence. For example, the effector RipS5 is encoded by a gene that has been clearly interrupted by a 34 kbp prophage. Table 6 also shows the orthologs of these genes in the related strains GMI1000, Po82, IPO1609, and UW551. In the table it can be seen that the genes that code for RipAA and RipAR have frameshifts or truncations in strain UY031 only. The absence of a particular effector may be enough for a pathogen to avoid host defenses, and therefore cause disease. These two genes are therefore a good starting point for additional investigations of phenotypic differences between these strains. Other effector genes of interest are those that are present and do not have disrupting mutations in UY031 but are absent or appear to be nonfunctional in other strains. We have found several such cases (Table 6), but in all cases there is at least one other strain that also has the same gene in what appears to be a functional state.

**Table 6:** List of T3E genes identified in *R. solanacearum* UY031 genome and their orthologs

Former effector name	New effector name^a^	UY031 (RSUY_)	GM1000 (RS)	Po82 (RSPO_)	IPO1609 (RSIPO_)	UW551 (RRSL_)
AWR2	RipA2	32720	p0099	m00080	03169	03418
AWR3	RipA3	40320	p0846	m01165	03901 + 05027^b^	-
AWR4	RipA4	40330/40^b^	p0847	m01166^b^	03902/3^b^	-
AWR5	RipA5_1	41860	p1024	m01289/90^b^	04049	01071
AWR5	RipA5_2	19780	-	c01821	01281	00546
Rip2	RipB	30390	c0245	c03161	00263	02573
Rip62	RipC1	42590	p1239	m01371	04123	03371
Rip34	RipD	33840	p0304	m01520	04484	00947
Rip26	RipE1	01190	c3369	c00070	03083	00852
-	RipE2	35100	-	c02513	04353	03923
PopF1	RipF1_1	45370	p1555	m01541	03403	04777
PopF2	RipF2	45510	-	m01557	05028/9^b^	04764
Gala2	RipG2	38790	p0672	m01007	04892	02264
Gala3	RipG3	32420	p0028	m00035	03202	00752
Gala4	RipG4	19910	c1800	c01835	01266/68^b^	00532
Gala5	RipG5	19920	c1801	c01836	01264	00531
Gala6	RipG6	17940	c1356	c01999	01463	01561
Gala7	RipG7	17950	c1357	c01998	01462	01562
HLK1	RipH1	19380	c1386	c01846	01319	00426
HLK2	RipH2	35470	p0215	m00201/2^c^	04317	03559
HLK3	RipH3	33320	p0160	m00157	03105	00041^b^
Rip1	RipI	00490 + 32050^b^	c0041	c03319	00098^b^	02976 + 02040^b^
Rip22	RipJ	24610^b^	c2132	c02749	-	-
Rip16	RipM	19180	c1475	c01871/2/3	01339 + 05024^b^	00705
Rip58	RipN	43290	p1130	m00869	04184	04736
Rip35	RipO1	34050	p0323	m01496	04463	00926
Rip63	RipQ	44390^b^	p1277	m00717	04287^b^	02855^b^
PopS	RipR	42640	p1281	m01376	04127	03375
SKWP1	RipS1	00860	c3401	c00036	00017	04182
SKWP2	RipS2	44630	p1374	m00690	04310	-
SKWP3	RipS3	41210	p0930	m01229	03993/4^b^	00237^b^
SKWP5	RipS5	10370 + 10840^b^	p0296	c02546^b^	-	-
SKWP7	RipS7	35110^b^	-	m00383	04352^b^	03921
Rip59	RipU	43920	p1212	m00805	04243	04660
Rip12	RipV1	17880	c1349	c02006	01470	01554
-	RipV2	19160^b^	-	c01875/76^b^	01341	00703
PopW	RipW	07010	c2775	c00735	02524	02682
PopA	RipX	40640	p0877	m01196	03933	02443
Rip3	RipY	30260	c0257	c03153	00276	01439
Rip57	RipZ	42040	p1031	m01312	04067	00271^b^
AvrA	RipAA	26380^b^	c0608	c02748	00659	01581
PopB	RipAB	40630	p0876	m01195	03932	02442
PopC	RipAC	40620	p0875	m01194	03931	02441
Rip72	ripAD	45790	p1601	m01585	03364	02518
Rip4	RipAE	29570	c0321	c03085	00343	01625
Rip41	RipAI	40230	p0838	m01156	03894	01021
Rip21	RipAJ	13300	c2101	c01332	04893	01260
Rip38	RipAL	39210^b^	-	m01053	-	02221
Brg40	RipAM	02270	c3272	c00191	02968	02810
Rip43	RipAN	40310	p0845	m01164	03900	01013
Rip50	RipAO	40750	p0879	m01206	03944	03105
Rip60	RipAP	43960	p1215^b^	m00800	04247	04655
Rip51	RipAQ	40810	p0885	-	03951	03113
Rip61	RipAR	44220^b^	p1236	m00770	04270	01136
Rip39	RipAV	39280	p0732	m01061	-	02213
Brg13	RipAX1	02040	c3290	m01221	02991	-
Rip55	RipAY	41810	p1022	m01283	04046	01066
-	RipBH	45880	-	m01600	03355	00782
-	RipBI	45200^b^	-	m00718	03419	00326
-	RipTPS	39290	p0731	m01062^b^	-	02212

^a^According to Peeters et al. [14]; ^b^: these genes appear to be nonfunctional due to various reasons (frameshift, truncation, etc.); genes in other columns that appear in the form locus tag x + locus tag y are genes which also appear to be nonfunctional due to frameshifts. ^c^:this gene is duplicated

Our modification analysis revealed two motifs that are essentially always methylated, namely: CAACRAC and GTWWAC. Both are fairly frequent in the genome, occurring respectively 2144 and 716 times. Motif CAACRAC is associated with the product of gene RSUY_11320 (R. Roberts, personal communication), which is hypothesized to be an enzyme of the Restriction-Modification System, with a restriction nuclease and a DNA methyltransferase role. This gene does not have homologs in other *R. solanacearum* strains and is located close to a region containing phage-related genes. This region contains gene RSUY_11410, which has been annotated as encoding a zonular occludens toxin. The provenance of this annotation is an enterotoxin gene found in *Vibrio**cholera* [23]; in *R. solanacearum* the role of this toxin gene is still unclear [24]. Motif GTWWAC is probably associated with the product of gene RSUY_22890 (R. Roberts, personal communication), which is hypothesized to be a solitary DNA methyltransferase (no restriction endonuclease linked). This gene does have homologs in other *R. solanacearum* strains (GMI1000, IPO1609, Po82 and PSI07). To our knowledge this is the first *R. solanacearum* genome with a methylome profile available.

## Conclusions

The complete sequence of *R. solanacearum* UY031 strain presented here should provide a rich platform upon which additional plant-pathogen studies can be carried out. Even though this is the fifth phylotype IIB1 sequenced, we found many differences with respect to the genomes of the other strains. In particular, the repertoire of T3E genes has many variations among these strains, and this may help explain some of the most relevant pathogenicity-related phenotypes described in the literature, opening the way to new control methods for bacterial wilt.

## Abbreviations

IIB1: Phylotype IIB, sequevar 1
T3E: Type III effectors
HGG: Homologous gene groups
